# Experience: developing an inpatient malnutrition checklist for children 6 to 59 months to improve WHO protocol adherence and facilitate quality improvement in a low-resource setting

**DOI:** 10.1080/16549716.2018.1503785

**Published:** 2018-08-09

**Authors:** Kathryn Beck, Angelique Mukantaganda, Scheilla Bayitondere, Richard Ndikuriyo, Almaque Dushimirimana, Vianney Bihibindi, Souzane Nyiranganji, Michel Habiyaremye, Jennifer Werdenberg

**Affiliations:** a Maternal and Child Health Department, Partners In Health/Inshuti Mu Buzima, Rwinkwavu, Rwanda; b Department of Pediatrics, Rwinkwavu District Hospital, Ministry of Health Rwanda, Rwinkwavu, Rwanda; c Boston Children's Hospital, Global Pediatrics Program, Massachusetts, USA

**Keywords:** Malnutrition, checklists, quality improvement, low-resource setting, under-5 mortality

## Abstract

In low-resource settings, inpatient case fatality for severe acute malnutrition (SAM) remains high despite evidenced-based protocols and resources to treat SAM. Key reasons include a combination of insufficiently trained staff, poor teamwork and inadequate compliance to WHO treatment guidelines which are proven to reduce mortality. Checklists have been used in surgery and obstetrics to ameliorate similarly complicated yet repetitive work processes and may be a key strategy to improving inpatient SAM protocol adherence and reducing unnecessary death. Here, we share our experience developing and piloting an inpatient malnutrition checklist (MLNC) for children 6 to 59 months and associated scoring system to coordinate care delivery, improve team documentation, strengthen WHO malnutrition protocol adherence and facilitate quality improvement in a district hospital in rural Rwanda. MLNC was developed after careful review of the 2009 Rwandan National Nutrition Protocol and 2013 WHO malnutrition guidelines. Critical steps were harmonized, extracted and designed into an initial MLNC with input from pediatric ward nurses, doctors, a locally based pediatrician and a registered dietitian. A scoring system was developed to facilitate quality improvement. Using the standard Plan-Do-Study-Act cycle, MLNC was modified and progress assessed on a monthly to bimonthly basis. Significant modifications occurred in the first 6 months of piloting including incorporation of treatment reminders and formatting improvements, as well as initiation of the MLNC from the emergency department. The MLNC is the first checklist to be developed that unifies WHO 10 steps of treatment of inpatient SAM with local standards. Anecdotally, MLNC was observed to identify gaps in key malnutrition care, promote protocol adherence and facilitate quality improvement. Data gathering on the MLNC local facility impact is underway. Collaborative international efforts are needed to create an inpatient malnutrition checklist for wider use to improve quality and reduce unnecessary, facility-based child mortality.

## Background

Globally, approximately 13 million children are diagnosed with severe acute malnutrition (SAM) annually, with 1–2 million preventable child deaths associated with SAM [1]. Evidence shows that WHO guidelines have led to improved survival and mortality of children admitted to inpatient facilities for SAM [2] and that case fatality may be reduced with strict compliance to treatment recommendations [3,4].

**Figure 1.**
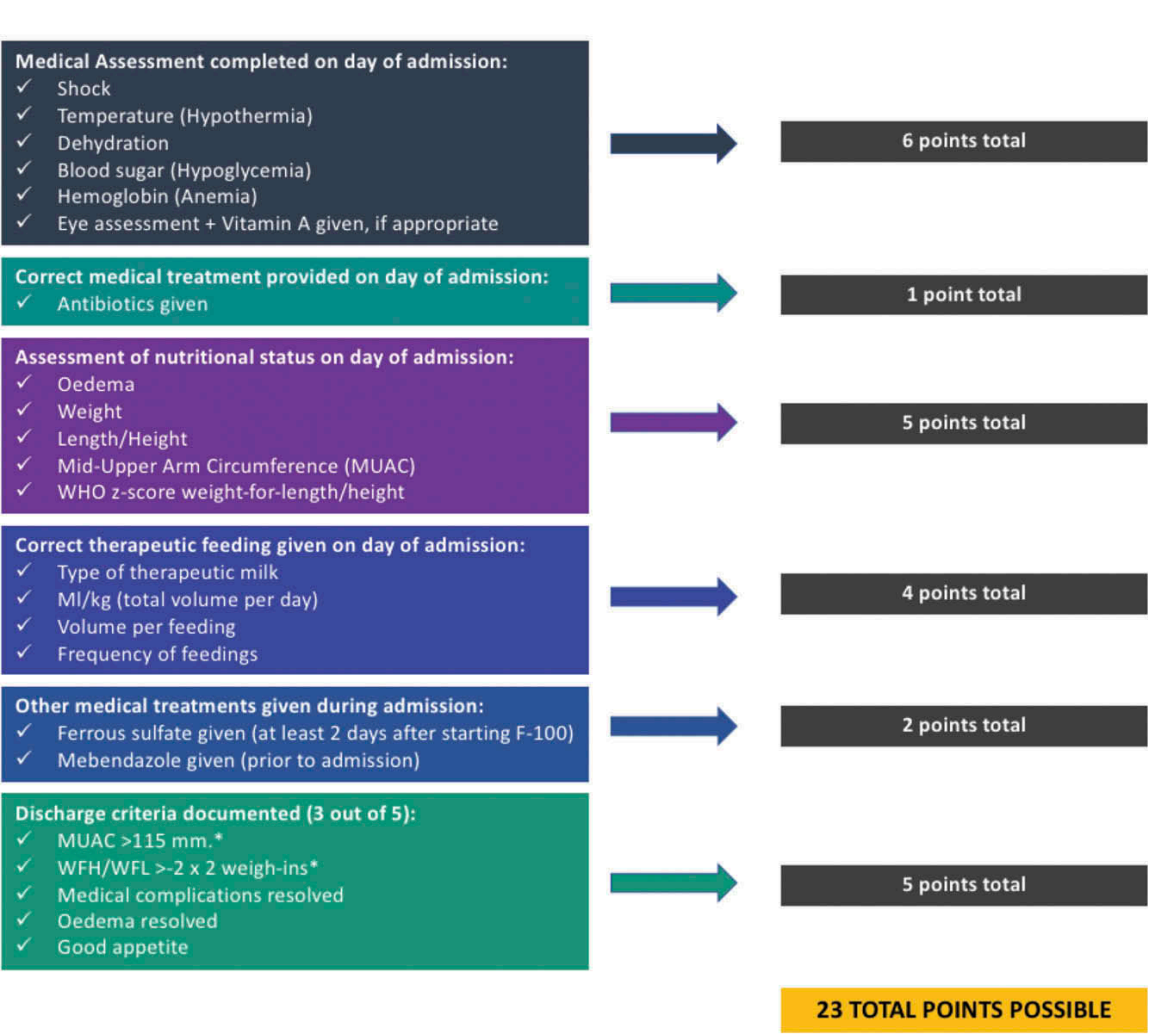
Scoring system for assessment of adherence to an inpatient malnutrition checklist (MLNC).

In low-resource settings, inpatient case fatality for inpatient malnutrition remains high despite availability of evidenced-based treatment protocols and resources [1,5]. Key reasons include insufficiently trained staff, poor teamwork and inadequate compliance to WHO treatment recommendations proven to reduce mortality [6–10]. WHO Checklists have been developed in surgery [11] and obstetrics [12] to ameliorate similarly complicated, yet repetitive work processes. The WHO Surgical Safety Checklist was created and implemented to decrease adverse events and increase teamwork amongst groups that can be hierarchical and disempowering to healthcare workers [13,14]. Since implementation, the Surgical Checklist has led to reductions in morbidity and mortality [15], as well as improved adherence to processes of care, teamwork and communication [14,15]. In anesthesia, checklists were able to improve process adherence and capture performance of critical safety elements [16].

In Rwanda, a national Performance-Based Financing (PBF) scheme was previously implemented to provide financial incentives to health providers for improvements in key national health priority output indicators related to maternal and child-care [17]. One of the indicators – ‘Number of malnourished children referred to district hospital for treatment during preventive care visit’ – is tied to improvement in higher-level referral for malnutrition. While this scheme may help to increase access to services via referrals, it’s limited in its ability to affect quality of inpatient malnutrition care.

We propose, that the development of a checklist to guide the inpatient management of malnutrition (encompassing severe and moderate complicated malnutrition as well as severe uncomplicated malnutrition) in low-resource settings is a key strategy to adherence improvement to the inpatient WHO malnutrition protocol and reduction of unnecessary deaths from malnutrition at the facility level. The inpatient management of acute malnutrition is particularly amenable to a checklist format, given 10 clear steps outlined by WHO. Progression of treatment steps is largely the same from patient to patient with most deviations during acute stabilization. Similar to surgery, malnutrition requires effective communication, timely action and collaborative team effort to ensure high quality, efficient patient care and reduce deaths. A malnutrition checklist could improve communication, streamline documentation and improve delivery of evidence-based malnutrition care to patients from the time of admission through rehabilitation and discharge to improve adherence to WHO protocols and reduce mortality.

Here, we describe development and pilot testing of an inpatient malnutrition checklist (MLNC) and scoring system for children 6–59 months. MLNC was developed to coordinate care delivery, team documentation, communication, improve inpatient WHO malnutrition protocol adherence and facilitate QI work on the wards of a district hospital in rural Rwanda. We hope that this shared experience and protocol to date can be used as a starting point for others to modify and utilize in their own settings. Additionally, we hope to raise awareness and call for an international collaborative to undertake the development of a global malnutrition checklist to be used as a resource in reducing child mortality.

## Objective

MLNC aimed to create a single, streamlined document that unified WHO and national Rwandan guidelines for inpatient malnutrition care that could be used by frontline healthcare workers as a guidance of critical steps in malnutrition management. MLNC was designed to coordinate an entire teams’ documentation of malnutrition care which can span weeks in duration. Thus, while initially filled out by a physician in the emergency department, the MLNC also includes documentation fields for nurses and/or nutritionists depending on workflow of the hospital. Lastly, a scoring system based on the MLNC was developed to enable hospitals to more easily implement quality improvement (QI) projects that identify and address gaps in key process indicators. In order to not disrupt regional requirements in medical record documentation, this checklist was implemented as additional rather than a replacement of routine documentation practices.

## Methods

A draft MLNC was developed in November 2015 after critical review of the Rwandan National Nutrition Protocol [18] and the WHO 2013 malnutrition guidelines [19]. There are 10 critical steps of inpatient acute malnutrition management from diagnosis in the emergency room through the completion of inpatient care [20]. These steps were extracted, harmonized, and designed into the initial MLNC with collaborative end-user feedback by local nurses and physicians, as well as a consultant pediatrician and registered dietitian-supporting treatment on the unit. Local staff, including the hospital’s general practitioners, pediatric and emergency room nurses, and nutritionist, were formally trained on MLNC theory and use to promote smooth adoption into routine clinical care.

Checklists were printed on colored paper to increase visibility in the patient chart and clean copies were kept in the emergency and pediatric departments. The emergency department doctor and nurse initiated the checklist, registered the child as having acute malnutrition, documented height and weight, and completed the initial medical management documentation. After transfer to the pediatric department, checklist documentation was reviewed and daily treatment iterated by the pediatric department staff (nurse, doctor, nutritionist). Generally, the nutritionist updated height, weight and calculated WHO z-scores; doctors prescribed feeding regimens and medications; and nurses documented care implementation and adverse reactions to feeding, such as vomiting. Pediatric team disagreements regarding care were addressed during ward rounds.

Over the next 6 months, experience with MLNC was discussed during weekly QI pediatric team meetings and revisions made based on user feedback. Last, a simplified scoring system was developed to assess malnutrition protocol adherence based on MLNC documentation (Supplemental Material Figure A: Malnutrition Checklist March 2017). Figure 1 describes the components of inpatient malnutrition management that were assessed for in the QI project and the associated scoring of the different components.

The pediatric QI team at Rwinkwavu District Hospital in Rwinkwavu, Rwanda began its first QI project using the MLNC in September 2015 focusing initially on documentation adherence. The subsequent QI project narrowed its scope to address specific documentation gaps, such as identification of shock in the emergency department for children with a diagnosis of SAM. The Plan-Do-Study-Act (PSDA) cycle of QI was used to plan the QI project, establish baseline indicators, implement use of the checklist, and make modifications as time went on.

## Results

MLNC was modified and progress of protocol adherence to the area of focus chosen assessed on a monthly to bimonthly basis. Run charts were displayed on the pediatric unit to track the progress of the QI project and to encourage staff

Modifications to MLNC during the first 6 months of use included clarifying criteria for advancing therapeutic milk feedings, prompts for medication provision on the appropriate day of treatment, medication dosage references, and guidance on discharge criteria and education. The nutritionist reported positive feedback related to the tool, particularly its ease in being able to monitor the daily progress of the patient, while the doctors felt that clearly outlining and prompting treatment for the 10 critical treatment steps was useful for proper treatment of malnutrition.

Now that MLNC has been in use for over two years, efforts are underway to document the QI project outcomes and its effects on inpatient malnutrition treatment processes. The QI teams continue to meet regularly and have begun identifying more granular components of the MLNCs to focus on, such as hypoglycemia.

## Conclusions

Preliminary experience with a locally developed MLNC suggests it is helpful in simplifying the complex treatment of inpatient malnutrition. Anecdotally, the MLNC was successfully implemented to address gaps in key malnutrition care processes as well as facilitate QI which promoted coordinated delivery of inpatient malnutrition services with improved adherence to protocols.

We experienced a number of challenges which will likely be common experiences to others using this tool or developing their own. First, the MLNC requires supportive implementation and leadership by respected mentors to facilitate the acceptance of the checklist and lead to improved team work. In our districts, we were fortunate that in addition to engaged hospital staff and regular support by a pediatrician and registered dietitian, hospitals are supported by routine QI mentorship through previously described MESH mentors [21–23] who provide enhanced supervision of staff on clinical and systems improvement initiatives. Second, the MLNC is ideally initiated in the emergency room setting requiring coordination between pediatric and emergency room staff. Poor early identification of malnutrition limits MLNC utility in the early, emergency management of acute malnutrition. Last, implementing MLNC as additional rather than replacement of current documentation puts a higher documentation burden on healthcare workers. However, as in our experience, once hospitals see the utility of MLNC they may choose to replace old documentation practices with the MLNC thus reducing redundancy in documentation and increasing visibility of malnutrition management.

In Rwanda, use of the MLNC has already expanded to a second District Hospital and we hope to share these experiences with other Rwandan facilities to improve the quality of care of inpatient acute malnutrition management regionally.

More broadly, we hope that this work will be the first step in calling for a MLNC which has input from international stakeholders and might be used and adapted to local country settings to improve adherence to evidence based protocols for inpatient malnutrition treatment, ultimately resulting in decreased mortality related to malnutrition for children under five.

## Paper context

Severe acute malnutrition remains a significant cause of preventable child death, despite clear protocols and resources to treat it. The World Health Organization has developed clinical checklists which have reduced facility based mortality. We developed and implemented an inpatient malnutrition checklist with a scoring system for documenting quality improvement. We hope this work will inspire international dialogue to create a global malnutrition checklist to promote quality care, World Health Organization protocol adherence and child survival.
