# Supplementary material for: Experience: developing an inpatient malnutrition checklist for children 6 to 59 months to improve WHO protocol adherence and facilitate quality improvement in a low-resource setting
Source: Glob Health Action. 2018 Aug 9;11(1):1503785. doi: 10.1080/16549716.2018.1503785 (PMC6097458; doi:10.1080/16549716.2018.1503785)
Supplement: Supplemental Material [file ZGHA_A_1503785_SM9365.pdf]

Ideal Weight for Discharge* (>-2 SD weight-for-height):________________

TREATMENT FOR ACUTE MALNUTRITION

Name of child Family name

Malnutrition classification: ❑ Severe uncomplicated ❑ Severe complicated ______________________________ ❑ Moderate complicated __________________________________

Day 1 (Admission):	**PHYSICAL ASSESSMENT ON ADMISSION**		
**(1)** Child unconscious or lethargic ❑ Yes ❑ No<br>Child's hands are cold ❑ Yes ❑ No<br>Slow capillary refill >3sec OR weak/fast pulse ❑ Yes ❑ No<br>**If yes to all three, doctor should begin treatment for SHOCK**	**(2) Temperature:** __________<br>❑ Axillary ❑ Rectal<br>**If axillary <35°C or rectal <35.5°C keep child warm**	**(3) Dehydration present:** ❑ Yes ❑ No<br>**Tolerating oral fluids** ❑ Yes ❑ No<br>If **YES** to both, start **oral rehydration;** If dehydration present **YES**, but **NO** oral fluids, start **IV rehydration**	**(4) Skin:**<br>❑ Dermatosis<br>❑ Skin Lesion<br>❑ Normal

**LABS & SCREENING ON ADMISSION**	**MEDICATIONS/VITAMIN/MINERAL TREATMENTS**		
**(5) Blood sugar ordered?** ❑ Yes **Result:** ___________ ❑ Unable to test<br>*Treat if hypoglycemia present: <54mg/dl OR, unable to test<br>**Treatment for hypoglycemia indicated** ❑ Yes ❑ No<br><br>**(6) Hemoglobin ordered?** ❑ Yes **Hemoglobin result:** ___________<br>*Give blood transfusion if Hb <4 g/dl or 4-6 g/dl with respiratory distress<br>**Transfusion indicated** ❑ Yes ❑ No→ **If YES, transfusion ordered** ❑ Yes ❑ No<br><br>**(7) TB symptom screen** ❑ Negative ❑ Positive **Symptoms:** ____________________<br>**(8) HIV test done** ❑ Yes	**(9) Vitamin A assessment**<br>Does the child have Bitot's spots, corneal clouding, corneal ulceration? ❑ Yes ❑ No<br><br>Has the child had measles <3 months?<br>❑ Yes ❑ No<br>**IF YES to either, vitamin A ordered**<br>❑ Yes ❑ No	**(10) Antibiotics**:<br>**Appropriate malnutrition antibiotics started**<br>❑ Yes<br>**see back for recommended empiric therapies*	**(11)** Other vitamin/mineral supplements **only necessary** when premixed F-75/F-100 or RUTF **are not used** (except iron, see back form)

FEEDING DAYS 1-7: Start with feed every 2 hours, including overnight; gradually increase to every 4 hours

***No pitting oedema/+1 pitting oedema: start 130 ml/kg/day *+2 pitting oedema/+3 pitting oedema: start 100 ml/kg/day**

*Increase volume/quantity to: 150 ml/kg (minimum) to 220 ml/kg (maximum) when appetite is good, reduced oedema, and no medical complications

		1	2	3	4	5	6	7
DOCTOR/NUTRITIONIST/NURSE	Date							
	Weight (kg)							
	Height (cm)							
	MUAC (mm)							
	WFH/L (weight-for-height)							
	Estimated amount vomited							
	Pitting oedema (0, 1+, 2+, 3+)							
	Formula/RUTF							
	Route of feeding							
	ml/kg or kcal/kg provided							
	Total daily volume (ml)/amount							
	Volume (ml)/amount per feed							
	Frequency							
	Antibiotics: ✓ if ordered							
	Vitamin A ***only if criteria met**	Dose given:	Dose given:					
	**Provider's initials**							

Ideal Weight for Discharge* (>-2 SD weight-for-height):________________

FEEDING DAYS 8-21: Feed every 4 hours, include night feedings
***No pitting oedema/+1 pitting oedema: start 130 ml/kg/day *+2 pitting oedema/+3 pitting oedema: start 100 ml/kg/day**
***Increase volume of feeds to: 150 ml/kg (minimum) to 220 ml/kg (maximum) when appetite is good, reduced oedema, and no medical complications**

		8	9	10	11	12	13	14	15	16	17	18	19	20	21
DOCTOR/NUTRITIONIST/NURSE	Date														
	Weight (kg)														
	Is MUAC **>115mm?**														
	Is WFH/L **>-2 SD?**														
	Est. amount vomited														
	Oedema (0, 1+, 2+, 3+)														
	Formula/RUTF														
	Route of feeding														
	ml/kg or kcal/kg provided														
	Total daily volume (ml)/amount														
	Volume (ml)/amount per feed														
	Frequency														
	Vitamin A: **if started**							Dose given:							
	Mebendazole: ✓ if given														
	Ferrous: ✓ when given Sulfate														
	**Provider's initials**														

MEBENDAZOLE: Received in the last 6 months?
❑ Yes ❑ No → If **NO**, give Mebendazole

Dose: 100 mg BID x 3 days
***Give only 7 days after admission**

FERROUS SULFATE: Give only when stable, gaining weight and in transition phase

Weight	Tablet	Syrup	Dose
3-6 kg	1/6 Tablet	0.5 ml	10 mg
6-10 kg	¼ Tablet	0.75 ml	15 mg
10-15 kg	¼ Tablet	1 ml	20 mg

VITAMIN A Dosing: Give if Bitot's spots, corneal clouding, or corneal ulceration OR, recent measles <3 months

Child's age	Vit A oral	Vit A IM
<6 months	50,000 IU	50,000 IU
6-12 months	100,000 IU	100,000 IU
>12 months	200,000 IU	100,000 IU

ReSoMal dosing loose stools: If +oedema 30 ml after each loose stool	Child's Age	Volume to give after each loose stool
	<2 years	50 – 100 ml
	≥ 2 years	100 – 200 ml

RECOMMENDED ANTIBIOTIC TREATMENT
Empiric therapies for UNCOMPLICATED SAM
1. Amoxicillin x 5 days

Empiric therapies for COMPLICATED SAM/MAM
1. **Ampicillin** x 2 days, followed by **Amoxicillin** x 5 days; Also start **Gentamycin** x 7 days at the same time that Ampicillin is given
2. **Ampicillin** + **Gentamycin** x 7 days
3. **Ceftriaxone** x 7 days
4. **Chloramphenicol** x 7 days

All patients:
- ✓ Does the child have a good appetite?
- ✓ Are all medical complications and oedema resolved?

If outpatient rehabilitation
- ✓ Did child consume 2 full meals of RUTF?

If inpatient rehabilitation
- ✓ Is the MUAC >115 mm?
- ✓ Is the WFH/L >-2 SD x 2 consecutive days? Is child at their ideal weight?

DISCHARGE: If answered yes to all questions, child may be ready for discharge

DISCHARGE EDUCATION:
- ❑ Counsel caregiver on good nutrition and cooking practices
- ❑ Instruct caregiver to immediately go to nearest health facility if child refuses to eat or has danger signs
- ❑ Instruct caregiver to follow-up in health center for admission to outpatient program
- ❑ Counter referral to health center
- ❑ If patient is receiving RUTF, give gap supply and contact health center to ensure stock
